# Dexamethasone Reduces Sensitivity to Cisplatin by Blunting p53-Dependent Cellular Senescence in Non-Small Cell Lung Cancer

**DOI:** 10.1371/journal.pone.0051821

**Published:** 2012-12-18

**Authors:** Haiyan Ge, Songshi Ni, Xingan Wang, Nuo Xu, Ying Liu, Xun Wang, Lingyan Wang, Dongli Song, Yuanlin Song, Chunxue Bai

**Affiliations:** 1 Department of Pulmonary Medicine, Zhongshan Hospital, Fudan University, Shanghai, China; 2 Department of Respiratory Medicine, The Affiliated Hospital of Nantong University, Nantong, China; Istituto di Ricerche Farmacologiche Mario Negri, Italy

## Abstract

**Introduction:**

Dexamethasone (DEX) co-treatment has proved beneficial in NSCLC patients, improving clinical symptoms by the reduction of side effects after chemotherapy. However, recent studies have shown that DEX could render cancer cells more insensitive to cytotoxic drug therapy, but it is not known whether DEX co-treatment could influence therapy-induced senescence (TIS), and unknown whether it is in a p53-dependent or p53-independent manner.

**Methods:**

We examined in different human NSCLC cell lines and detected cellular senescence after cisplatin (DDP) treatment in the presence or absence of DEX. The *in vivo* effect of the combination of DEX and DDP was assessed by tumor growth experiments using human lung cancer cell lines growing as xenograft tumors in nude mice.

**Results:**

Co-treatment with DEX during chemotherapy in NSCLC resulted in increased tumor cell viability and inhibition of TIS compared with DDP treated group. DEX co-treatment cells exhibited the decrease of DNA damage signaling pathway proteins, the lower expression of p53 and p21^CIP1^, the lower cellular secretory program and down-regulation of NF-κB and its signaling cascade. DEX also significantly reduced DDP sensitivity *in vivo*.

**Conclusions:**

Our results underscore that DEX reduces chemotherapy sensitivity by blunting therapy induced cellular senescence after chemotherapy in NSCLC, which may, at least in part, in a p53-dependent manner. These data therefore raise concerns about the widespread combined use of gluocorticoids (GCs) with antineoplastic drugs in the clinical management of cancer patients.

## Introduction

Glucocorticoids (GCs) such as dexamethasone (DEX) are widely used as a co-treatment with chemotherapy because of their effectiveness to prevent edema, nausea, and toxic reaction caused by cytotoxic treatment in tumor patients [1]. GCs co-treatment also improves appetite, weight loss and relieves bone pain [2]. However, recent studies indicate that GCs can inhibit the effect of chemotherapy not only in established cancer cell lines and tumor xenografts [3], [4], [5], but also in the freshly isolated cells from surgical resections from tumors of various origins [6], [7], [8]. Moreover, GCs co-treatment reduces several chemotherapy drugs sensitivity, including DDP [3], paclitaxel [9], [10], trastuzumab [4], gemcitabine [6] and camptothecin [11]. A growing body of evidence suggested that GCs reduced cellular sensitivity to chemotherapy may occur through multiple mechanisms, for example, by enhancing DNA repair capacity, suppressing host anti-tumor immune responses, and blocking apoptosis [5], [11], [12]. However, the molecular mechanisms underlying the effect of GCs are still largely unknown.

Worldwide, lung cancer counts for the largest number of new cases of cancer and deaths from cancer annually [13], and a large amount of evidence indicate that DDP-based adjuvant chemotherapy improves survival among patients with completely resected non-small-cell lung cancer [14], [15], [16]. Cytotoxic drugs such as DDP can activate DNA damage signaling pathway [14], [17] and the sensing proteins, such as γH2AX and 53BP1, which are instrumental in driving cellular senescence [18], [19]. Cellular senescence is characterized by an irreversible arrest of cell proliferation, so that it can prevent the aberrant and unlimited proliferation of tumor cells [20]. Senescent cells exhibit enlarged morphological changes and less motility than young cells, which may contribute to the suppression of cell migration, invasion, and metastasis [21]. Therefore, cellular senescence acts as an important barrier to cancer and plays an important role in tumor suppression.

To analyze whether DEX might also reduce DDP sensitivity by blunting cellular senescence, we examined cellular senescence in different human NSCLC cell lines and xenografts after DDP treatment in the presence or absence of DEX. We found that DEX had a strong anti-senescence effect in the used carcinoma cells as well as in human xenografts in nude mice. This was because of the inhibition of NF-κB activity resulting in a blockade of p53 signaling pathway. Direct transfer of p53 and NF-κB restored senescence sensitivity of DEX-treated carcinomas in NSCLC cell lines. These *in vitro* and *in vivo* data suggest a need for carefully considering the use of DEX and other GCs together with cytotoxic therapy in the treatment of patients with lung carcinoma.

## Materials and Methods

### Cell Cultures and Stimulation of Cells

NSCLC cell lines A549, NCI-H292 and NCI-H1299 were purchased from the Cellular Institute of Chinese Academy of Science (Shanghai, China) [22], [23], [24]. A549 cells were grown in F12-K medium. H292 and H1299 cells were cultured in RMPI 1640 medium. DDP (Sigma Aldrich) was dissolved in DMF at a concentration of 10 mM. A 10 mM stock of DEX (Sigma Aldrich) was prepared in double distilled water. After pretreatment of DEX for 24 hours, cells were treated with DDP for 48 hours. Cells were then washed to remove drugs and incubated for additional 3 days in fresh media.

### Proliferation Screening and Cell Proliferation Assay

The number of viable cells was estimated using the Cell Counting Kit-8 (Dojindo, Kumamoto, Japan) assay which provided effective and reproducible determination of proliferative activity of cells. To measure the proliferative activity of cells in 96-well microplates, CCK-8 was added (10 µl/well) and incubation continued for 1 hour. Absorbance was measured at 450 nm using a microplate reader (Molecular Devices) with a reference wavelength of 650 nm.

### Alive Measurement of Cell Bio-behaviors

Cell bio-behaviors were measured by real-time cell monitoring system, using a Cell-IQ cell culturing platform equipped with a phase-control microscope and a camera [25]. Images were captured at 5 minutes intervals for 48 hours. Analysis was carried with a freely distributed Image software (McMaster Biophotonics Facility, Hamilton, ON, Canada), using the Manual Tracking plugin created by Fabrice Cordeliéres (Institut Curie, Orsay, France). Treated with DDP in the presence or absence of DEX, then A549 and H292 cells were cultured in Cell-IQ system with 24 well plates for 48 hours. Cell-IQ system automatically discriminated cell stage and calculated total cell numbers. Each group contained 6 replicate image sites.

### Immunofluorescence

Cells were fixed in 4% paraformaldehyde for 15 min and permeabilized in PBS-0.2% Triton for 10 min. After blocked for 1 hour, primary antibodies (γH2AX: 2212-1, epitomics, 1∶100; 53BP1: AB36823, Abcam, 1∶100, NF-κB: Cell Signaling Technology, 1∶100) were diluted in blocking buffer and incubated with fixed cells overnight at 4°C. Cells were washed, incubated with secondary antibodies (Jackson, 1∶100) for 1 hour at room temperature. All slides were counterstained with 4′,6-diamidino-2-phenylindole (DAPI). Immunofluorescence was performed using confocal laser scanning microscopy (Lecia) or fluorescence microscopy (Olympus).

### Apoptosis Assays, BrdU Incorporation

The TUNEL reaction was performed using the TUNEL in situ cell death detection kit-fluorescein (Roche applied science, Laval, Quebec, Canada) according to manufacturer’s instructions. Briefly, cells were fixed in 4% paraformaldehyde for 10 min. Cells were then permeabilized for 2 min on ice before labeling with 50 µl of TUNEL reaction mixture and incubating at 37°C for 1 hour. After washed, slides were mounted and examined by fluorescence microscopy. The percentage of apoptotic cells was calculated as (TUNEL-positive cells/total cells)×100%. Incorporated BrdU was detected by the BrdU cell proliferation assay (QIA58, Calbiochem, Merck, Germany). BrdU was added and incubated for an additional 24 hours, and then fixative/denaturing solution was added for 30 min. Anti-BrdU antibody (1∶100) was added to interact with incorporated BrdU for 1 h at room temperature. Then, cells were incubated with anti-BrdU antibody (1∶1000) for 30 min. After washed, stop solution were added and absorbance was measured using a spectrophotometric plate reader at dual wavelengths of 450–540 nm.

### Senescence-associated β-galactosidase (SA-β-gal) Staining and Quantification

Cells were washed to remove drugs and incubated for an additional 3 days in fresh media. In situ staining of SA-β-gal with cells or frozen sections were performed using a senescence-β galactosidase staining kit (Beyotime Institute of Biotechnology, China) following the manufacturer’s instructions. Cells were considered positive when the cytoplasm was stained with SA-β Gal. All experiments were performed in triplicate.

### FACS Analysis

Cells were fixed in 70% ice-cold ethanol and stained with PBS containing 50 µg/ml propidium iodide (Sigma-Aldrich) and 100 µg/ml RNase A for DNA content analysis by flow cytometry analysis on a FACSCalibur system (BD). The percentage of cells in various cell cycle phases was calculated using FlowJo software (Tree star Inc., Ashland, OR, USA).

### RNA Extraction and Real-time PCR

Total RNA was extracted using the RNeasy minikit (QIAGEN, Hamburg, Germany). RNA (2 µg) was reversely transcribed to complementary deoxyribonucleic acid and 40 ng of complementary DNAs were used as a template for real-time PCR. The amplification cycling reactions (40 cycles) were performed as follows: 5 secs at 95°C and 30 secs at 60°C. Relative quantification values of the target genes were standardized according to the comparative threshold cycle (2^−ΔΔCT^).

### Western Blot Analysis

Proteins (30 µg) in the total cell lysate were separated by SDS-PAGE and transferred to nitrocellulose membranes. Membranes were blocked for 1 hour at room temperature and washed 3 times with 15 ml of TBS/0.1% Tween. Blots were then incubated with anti-PCNA (1∶1000; Millipore), anti-triMe H3K9 (1∶1000, Cell signaling), anti-triMe H3K27 (1∶1000, Cell signaling), anti-53BP1 (1∶1000; Abcam), anti-γH2AX (1∶1000; Epitomics), anti-PCNA (1∶1000; Sigma-Aldrich), anti-p53 (1∶1000, Cell Signaling), anti-p21^CIP1^ (1∶1000; Cell Signaling), anti-p27^KIP1^ (1∶1000, Cell signaling), anti-pRb (1∶1000, Cell signaling), anti-Cyclin A (1∶1000; Cell Signaling) and anti-GAPDH (1∶1000, Sigma-Aldrich). Membranes were washed and then incubated with secondary antibody (1∶1000) for 1 hour. Bands were visualized by chemiluminescence (ECL; Amersham) followed by exposure to x-ray film (RX-U; Fujifilm). Bands density was densitometrically quantified using ImageJ (National Institutes of Health).

### Luciferase Reporter Assays

The pGL3-p53 and pGL3-NF-κB firefly luciferase reporter plasmids (Gift from Dr HO Liu) were used in conjunction with the control pGL3-basic vector (Promega) and internal control plasmid pRL-SV40 (Promega). Cells were cultured in 24-well plates 1×10^5^/well overnight and transfected with the indicated plasmid using X-tremeGENE HP DNA Transfection Reagent (Roche, 06366236001) according to the manufacturer’s instructions. Cells were harvested 24 hours after transfection and lysates were analyzed for firely and renilla luciferase activity using the Dual Luciferase Reporter Assay System (Promega, Madison, WI). Data were represented as the fold induction compared to the pGL3-Basic vector. Three independent transfection experiments were performed in triplicate for each experimental construct.

### Nude Mice and Xenografts

Our animal experiment was approved and carried out in strict adherence to the policies and guidelines set forth by FuDan University Animal Care and Use Committee. Briefly, 1×10^7^ A549 cells with 100 µl PBS were injected to the flank of 4-week-old nude mice. DDP 5 mg/kg was injected intraperitoneally weekly over a period of 4 weeks when the tumor sizes reached a volume of 200 mm^3^ in the absence or presence of DEX. DEX 0.2 mg/kg was given by gavage twice daily the day before, the day of, and the day after DDP administration. Tumor growth was serially monitored with calipers measurement of two perpendicular diameters. Tumor volume was calculated using the formula: 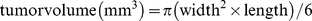. Mice were humanely euthanized at tumor sizes >3000 mm^3^.

### Statistical Analysis

All results were expressed as mean ± SEM. Group differences were evaluated by one-way ANOVA or by Student’s *t*-test as appropriate. Kaplan-Meier analysis was used to calculate survival probabilities. Differences between experimental groups with *P*<0.05 were considered statistically significant.

## Results

### DEX Co-treatment Increased Cell Viability in DDP Treated NSCLC Cell Lines

**Figure 1:**
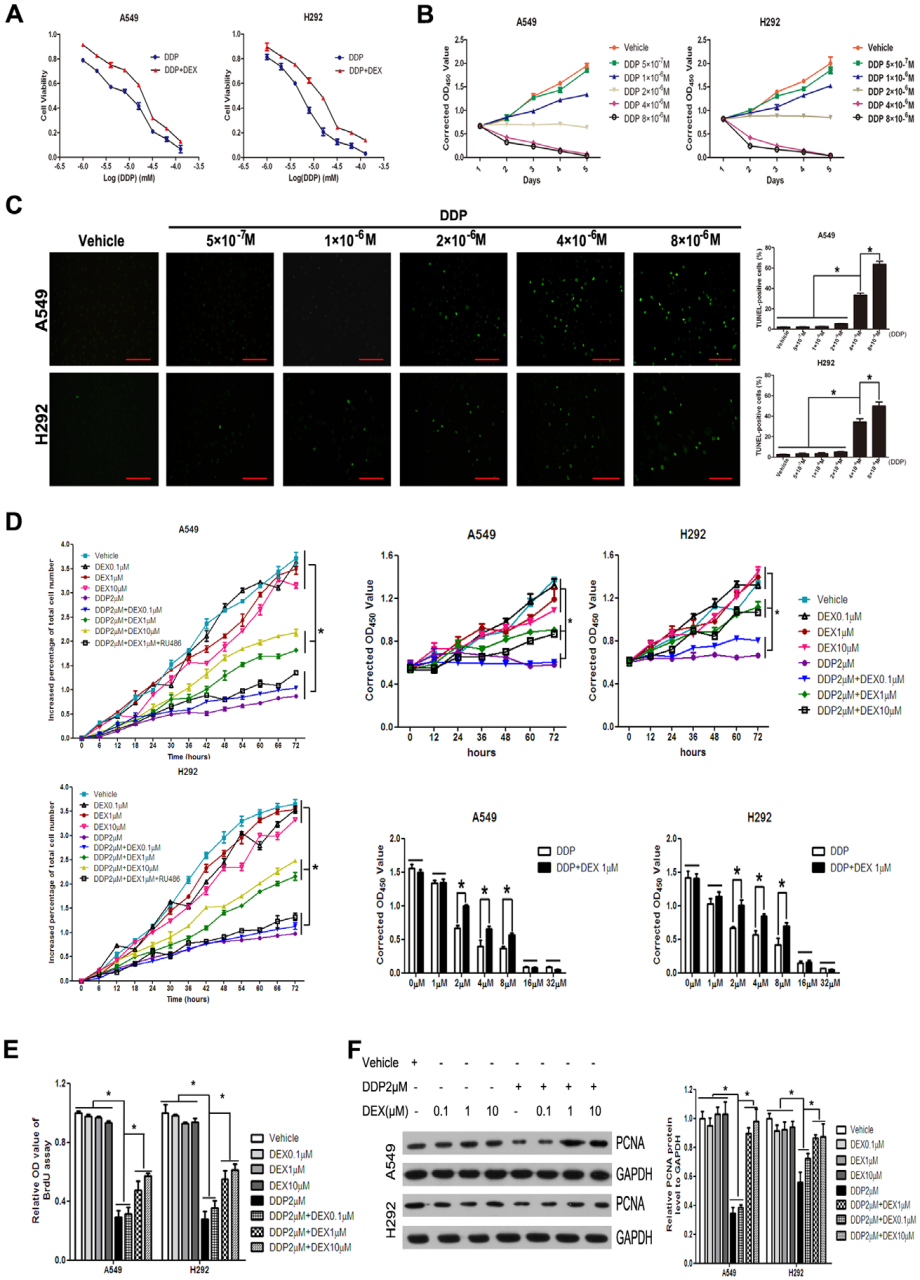
DEX protected cell survival in response to cytotoxic drugs in non-small cell lung cancer cells. (A) The detection of IC50 by CCK-8 assay. NSCLC cell lines were cultured in a medium containing various doses of DDP (0 µM, 1 µM, 2 µM, 4 µM, 8 µM, 16 µM, 32 µM, 64 µM, 128 µM) as indicated in the absence or presence of 1 µM DEX for two days, and then the viability of cells was monitored. (B) Cell viability assay in response to the indicated DDP concentrations (0 M, 5×10^−7^ M, 1×10^−6^ M, 2×10^−6^ M, 4×10^−6^ M and 8×10^−6^ M) was measured by CCK-8 assay for 5 days, including 2 days of DDP treatment and 3 additional days of drug free medium incubation. (C) Representative fluorescence microscopic images of A549 and H292 cells after TUNEL assay (left) and quantitative analysis of apoptosis (right). (D) The detection of increased percentage of total cell numbers by Cell-IQ and CCK-8. Total cell numbers of A549 and H292 were measured 72 hours after cultured with different drugs. (E) BrdU incorporation of A549 and H292 cells. BrdU was incubated for 24 hours, and then cells were fixed and detected with BrdU. For quantity analysis of BrdU incorporation, relative OD values were assayed compared with vehicle group. (F) Western blot analysis showed PCNA protein levels in A549 and H292 cells and quantitative data of PCNA protein levels. Data represent means of six determinations ± standard error of mean (SEM). *p<0.05 and **p<0.01 analyzed by one-way analysis of variance (ANOVA).

In order to investigate the effects of DDP alone or plus DEX on the survival *in vitro*, we used CCK8 assay and examined in NSCLC cell lines A549 and H292. DDP could significantly decrease the survival cell numbers in a dose-dependent manner in NSCLC cells. In A549 and H292 cell lines, the IC50 was 7.12 µM and 5.44 µM, respectively. Co-treatment with 1 µM DEX significantly increased the survival cell numbers and IC50 (A549∶15.78 µM, H292∶11.60 µM) compared with DDP alone group (*P*<0.05) (Figure 1A).

Incubation of the cell lines A549 and H292 cells with DDP for 2 days followed by a drug free medium for 3 days. Depending on DDP concentrations, proliferation arrest and/or cell death occurred as a result. We chose six different concentration gradients of DDP (0 M, 5×10^−7^ M, 1×10^−6^ M, 2×10^−6^ M, 4×10^−6^ M and 8×10^−6^ M) and examined with CCK-8 assay. At concentrations less than 2×10^−6^ M, DDP delayed cellular proliferation, and at 2×10^−6^ M, the cell number remained constant during the entire period of incubation suggesting irreversible growth arrest, which meant senescence. In contrast, greater DDP concentrations induced cell death (Figure 1B). To confirm this effect, we also chose the above six different concentration gradient of DDP and evaluated in A549 and H292 cells with TUNEL assay. Treatment with DDP which concentrations less than or equal to 2×10^−6^ M did not show any significant change, whereas DDP which concentrations greater than 2×10^−6^ M significantly increased the percentage of TUNEL-positive cells (Figure 1C). Therefore, we used 2×10^−6^ M DDP in which concentration can induce cellular senescence rather than apoptosis in the following experiment.

Next, we assessed the effects of DEX co-treatment on the percentage of total cell numbers with Cell IQ in A549 and H292 cell lines. The percentage of total cell number significantly decreased after the stimulation of DDP as compared to vehicle. DEX co-treatment increased the total cell number by 17.21%, 15.51% (0.1 µM DEX, A549, H292, respectively), 95.06%, 118.43% (1 µM DEX, A549, H292, respectively), 131.18%, 150.11% (10 µM DEX, A549, H292, respectively) in the presence of DDP (Figure 1D). Since concentration of 1 µM DEX or higher can significantly increase the total cell number, we used 1 µM DEX in the following experiment. Pretreatment with glucocorticoid receptor (GR) antagonist, RU486, could block the influence of DEX, which suggest that DEX reduced DDP sensitivity was by acting through the GR. Besides, we also performed CCK-8 assay and confirmed DEX comedication role (Figure 1D). The efficiency of different doses of DDP(1 µM, 2 µM, 4 µM, 8 µM, 16 µM, 32 µM) in the presence or absence of DEX was also detected by CCK-8. DEX co-medication indicated protective effects at different doses of DDP (2 µM, 4 µM, 8 µM) and ineffectiveness when dosages exceeded 16 µM (Figure 1D).

To complement the above finding, we next examined cell proliferation with BrdU, a thymidine analogue which can incorporate into the newly synthesized DNA of S-phase. DDP decreased normal proliferation of A549 and H292 cells whereas DEX attenuated this decrease (Figure 1E). Furthermore, we also detected the expression of proliferating cell nuclear antigen (PCNA), a protein involved in DNA replication and DNA repair, through Western-blot analysis. DDP also decreased the protein expression of PCNA, and combined DEX treatment can reduce this decrease (Figure 1F). These data suggest that DEX co-treatment increase cell viability in DDP treated NSCLC cell lines.

### DEX Protected Tumors by the Inhibition of TIS

**Figure 2:**
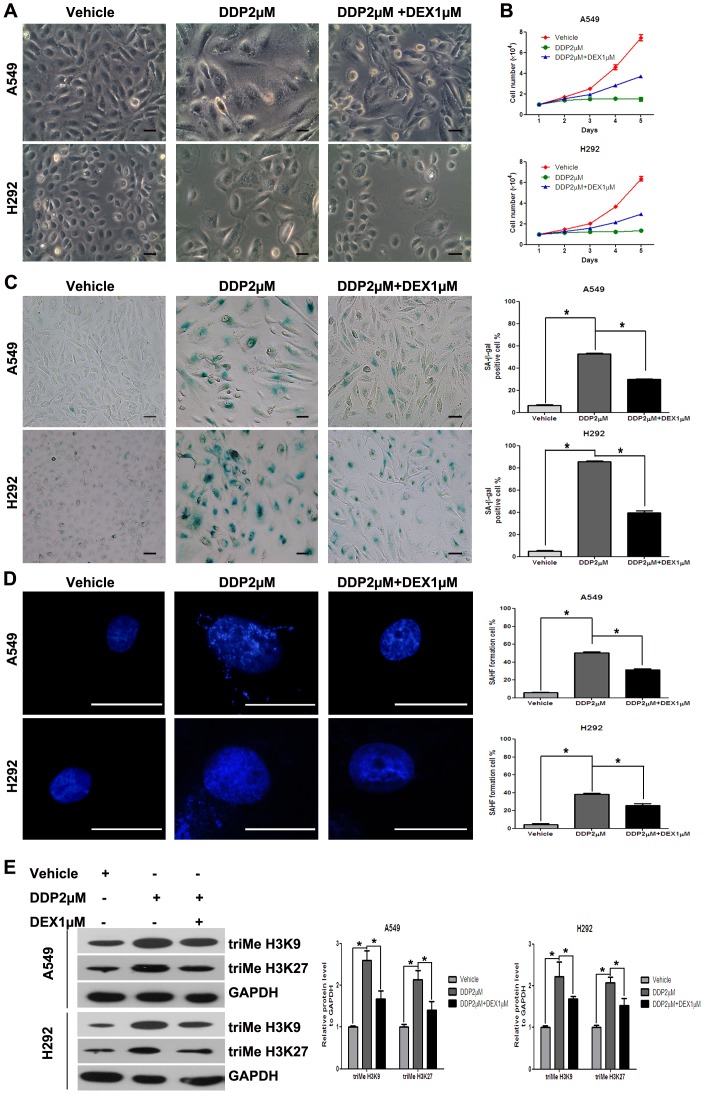
DEX protected tumors by the inhibition of TIS in A549 and H292 cells. (A) Cells were cultured in drug-free medium, fixed, and stained. Cells were visualized under ×200 magnification using phase contrast microscopy. (B) Cell proliferation assay was performed after drug wash, and cells were counted for 5 days. (C) SA-β-gal activity was analyzed by microscopy at day 3 after drug wash (left). The percentage of SA-β-gal-positive cells was presented in the histogram (right). (D) Representative photos for SAHF formation in A549 and H292 cells at day 3 after drug wash. SAHF formation was quantified by counting 200 cells from >10 random fields, and the results were shown in the right histogram. (E) Proteins of A549 and H292 cells were incubated with antibodies specific for trimethylated lysines H3K9 and H3K27 and analyzed by western bolt. Expression of GAPDH was taken as an internal loading control. Data represent means of six determinations ± standard error of mean (SEM). *p<0.05 and **p<0.01 analyzed by one-way analysis of variance (ANOVA).

Since 10 µM DEX alone led to a slight inhibition of cell growth in A549 and H292 cells (Figure 1D), the increase in survival cell number when cells were co-treated with DEX and DDP was not due to the acceleration of cell growth, but most probably was due to influence TIS. To test this probability, we further examined the change in cellular senescence of A549 and H292 cells with DDP or DEX alone or with a combination of DEX and DDP for 48 hours. And we identified senescent cells on the basis of multiple characteristics. First, we noted that DDP caused remarkable and characteristic morphological alterations, including enlarged cellular size and a flattened shape in A549 cells and H292 cells, and DEX co-treatment attenuated these morphological alterations (Figure 2A). DEX co-treatment also significantly affect the growth of A549 and H292 cell lines (Figure 2B).

Next, we detected a widely accepted marker, SA-β-gal activity, which stains the perinuclear compartment blue. DDP significantly induced increased SA-β-gal activity, and interestingly, DEX co-treatment decreased SA-β-gal activity compared with that of DDP treatment alone (Figure 2C). Furthermore, we also observed obvious senescence-associated heterochromatin foci (SAHF) formation, which is thought to be a senescence nuclei biomarker, in DDP treated A549 and H292 cells. The percentage of SAHF-positive cells was significantly increased with the DDP treatment. DEX co-treatment could decrease the percentage of SAHF-positive cells compared with that of DDP treatment alone (Figure 2D). The composite foci of SAHF contain methylated and deacetylated histones and other associated proteins. Widely tested markers in this category include methylation of histone 3 at lysines 9 and 27 and phosphorylation of H2AX histone family, member X (γ-H2AX), all of which colocalize in SAHF [19], [26]. We detected the expression of triMe H3K9 and triMe H3K27 and the results indicated the same trend of SAHF (Figure 2E).

### DDP-induced DNA Damage was Inhibited in the Presence of DEX

**Figure 3:**
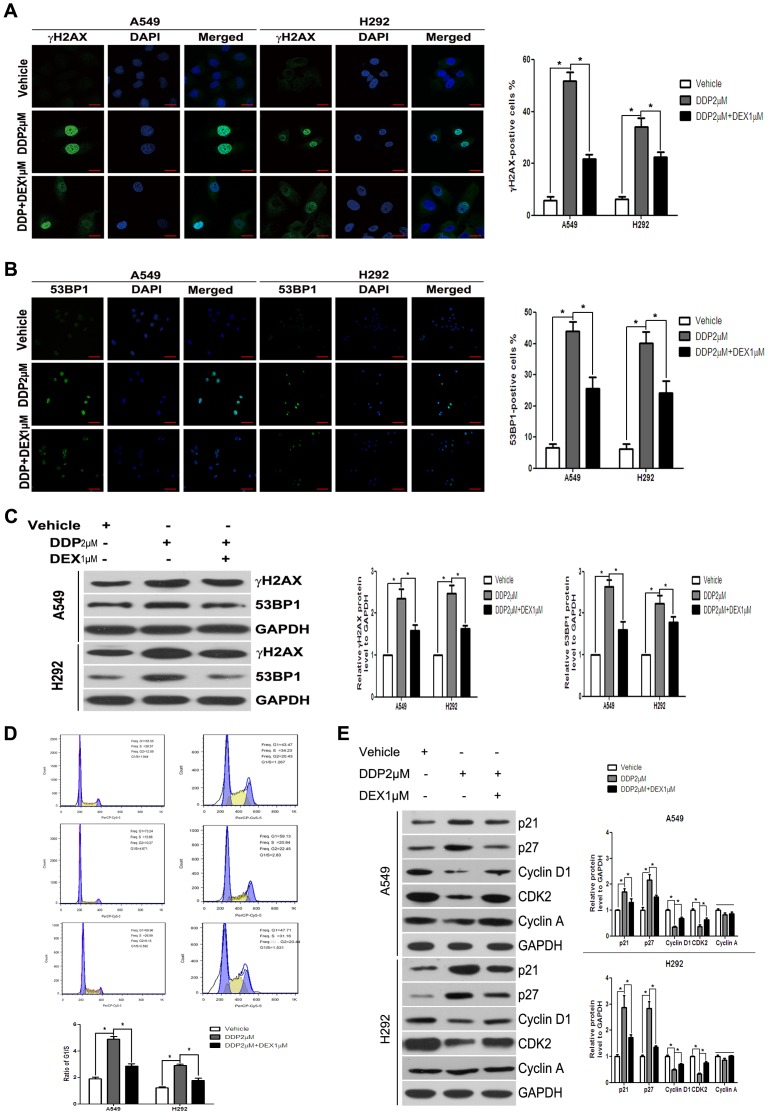
DEX protected tumors by the inhibition of TIS in A549 and H292 cells. (A) Cells were fixed and stained for γH2AX foci (green) 48 h after drug wash. DAPI staining was performed to visualize the nuclei (blue). Percentage of γH2AX-positive cells is presented in the histogram (right). (B) Cells were fixed and stained for 53BP1 foci (green) 48 hours after drug wash. DAPI staining was performed to visualize the nuclei (blue). Percentage of 53BP1-positive cells is presented in the histogram (right). (C) After DDP treatment for 48 hours in the presence or absence of DEX, cells were washed and cultured for another 48 hours. γH2AX and 53BP1 protein level were investigated by Western blot analysis. Expression of GAPDH was taken as an internal loading control. (D) Cell cycle analysis was performed at 48 hours after drug wash. The percentages of G1, S and G2 are demonstrated as shown. The histogram displays the relative changes of G1 phase compared with S phase. (E) Expressions of p21, p27, Cyclin D1, CDK2 and Cyclin A protein level were investigated by Western blot analysis. Expression of GAPDH was taken as an internal loading control. Data represent means of six determinations ± standard error of mean (SEM). *p<0.05 and **p<0.01 analyzed by one-way analysis of variance (ANOVA).

Because DEX influences therapy-induced and replicative senescence, and DNA-damage response signaling plays a core role in both, we next investigated whether DEX modulates the DNA damage checkpoint. We monitored therapy-induced DNA damage by examining two commonly used markers of DNA damage, γH2AX and 53BP1. DDP treated cells, as expected, had an increase in DNA damage over control cells, and DEX co-treatment with DDP attenuated the degree of DNA damage, as judged by either γH2AX or 53BP1 (Figure 3A, 3B, and 3C).

Senescent cells never reenter the cell cycle and appear to decrease in DNA synthesis, growth arrest is achieved and maintained in either G1 or G2/M stage of the cell cycle, in part by the increased expression of specific cyclin-dependent kinase inhibitors (CDKIs). We performed FACS analysis and found DDP therapy induced cell cycle arrest at G1 phase, accompanied by the decrease in percentages of S phase, and DEX decreased the degree of these changes (Figure 3D). Detections of senescence-associated cell cycle regulatory proteins discovered up-regulations in p21 and p27, and down-regulations in Cyclin D1 and CDK2 in DDP-treated cells, and correspondingly weakened trends in DEX co-treated cells (Figure 3E). These results supported the assumption of DEX’s influence over DNA damage checkpoint. These results are consistent with DEX affecting senescence by influencing the activation of the DNA damage checkpoint.

### DEX Co-treatment Reduced DDP Sensitivity in a p53-dependent Manner

**Figure 4:**
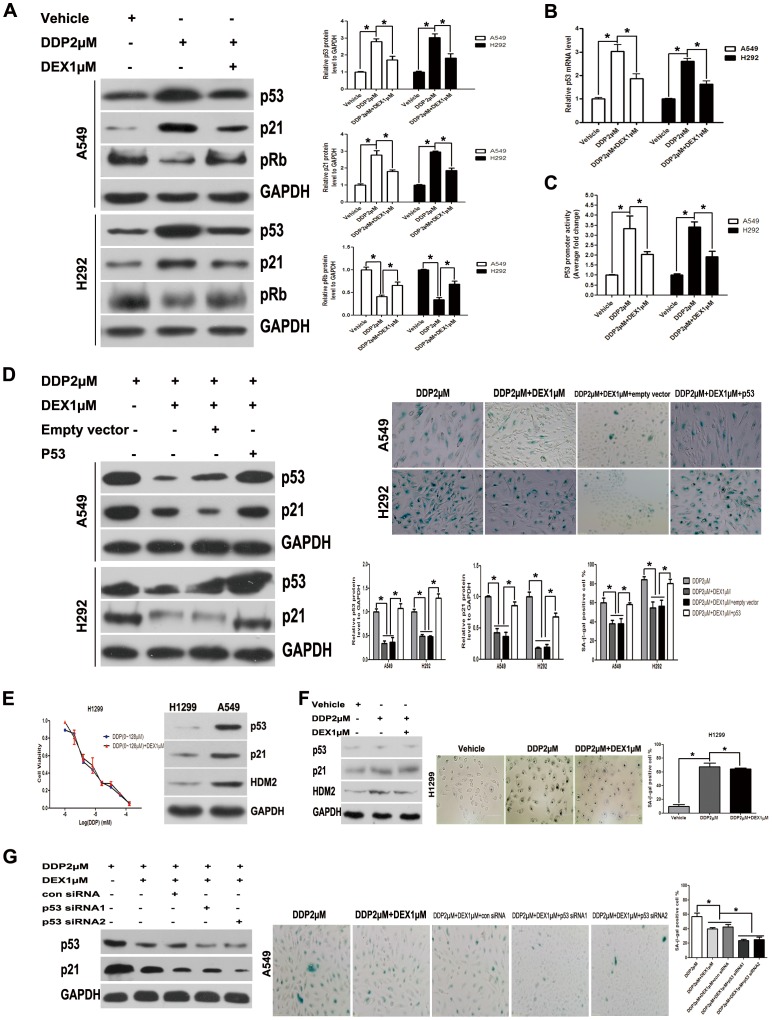
Co-treatment reduced DDP sensitivity in a p53-dependent manner. (A) The p53, p21 and pRb in lysates made from A549 and H292 cells was determined by Western-blot, equivalent protein loading between lanes was confirmed by Western analysis of GAPDH levels. (B) Changes of mRNA expression levels of p53 after DDP treatment in the presence or absence of DEX were detected by quantitative real time PCR. Fold changes were calculated from β-actin normalized Ct values. (C) Cells were transiently transfected with p53 promoter-luciferase reporter plasmid. The luciferase activity in whole cell lysates was determined 24 hours post-transfection. Data shown are mean (± standard error of mean) of the ratio firefly luciferase activity over renilla luciferase activity and normalized to the ratio of vehicle group. (D) A549 and H292 cells transfected with a p53 expression vector (pcDNA3.1-/p53) were treated with DDP in the presence or absence of DEX. SA-β-gal activity was analyzed by microscopy at day 3 after drug wash. Percentage of SA-β-gal cells was presented in the histogram. (E) H1299 (p53 deficient type) cells were used to detect whether DDP insensitivity occurred in p53 dependent manner by CCK-8 assay using various doses of DDP (0 µM, 1 µM, 2 µM, 4 µM, 8 µM, 16 µM, 32 µM, 64 µM, 128 µM). The p53 statuses were confirmed by examining p21^CIP1^ and hdm2 expressions. (F) SA-β-Gal activity was detected in H1299 cells treated with DDP in the presence or absence of DEX. (G) The effect of siRNAs designed against p53 was measured by western-blot in A549 cells. SA-β-Gal activity was detected and analyzed. Data represent means of six determinations ± standard error of mean (SEM). *p<0.05 and **p<0.01 analyzed by one-way analysis of variance (ANOVA).

The p53 pathway is important for establishing and maintaining the senescence growth arrest caused by cytotoxic stress. We therefore asked whether DEX co-treatment influenced chemotherapy sensitivity by p53 pathway. After DDP treatment, p53 protein gradually accumulated, and effect was strikingly weakened in the presence of DEX. The same trend was also detected about the protein expression of p21^CIP1^, a well-established transcriptional target and downstream effector of p53 with functions in cell cycle arrest, senescence induction and apoptosis (Figure 4A). Strong induction of p21 was expected to inhibit the activity of G1 cyclin/CDK complexes to result in hypophosphorylation of retinoblastoma protein (pRb) and induction failure of S phase cyclins (Figure 4A). Similarly, p53 mRNA level was also increased after DDP treatment, and DEX co-treatment attenuated this increase (Figure 4B). Furthermore, we also used luciferase reporter assays to detect p53 promoter activity, and the analysis showed DEX co-treatment could also attenuate p53 promoter activity compared with that of DDP treated group (Figure 4C). These changes corroborated the G1 phase retention observed in DNA histograms and remained consistent with the irreversible G1 arrest observed in chemotherapy-induced senescence. Thus, different biological outcomes of DDP treatment in the absence or presence of DEX arose from differential regulation of p53 and its downstream target p21.

To further confirm the effect of p53/p21^CIP1^ pathway, A549 and H292 cells were treated with pcDNA3.1-/p53. After 2 days of DDP treatment with or without pcDNA3.1-/p53, we investigated that overexpression of p53 could prevent the decrease of SA-β-Gal activity in DEX co-treatment group (Figure 4D). Furthermore, to analyze whether DEX-associated DDP insensitivity depended upon co-treatment reduces DDP sensitivity is p53 dependent, we used two cell lines differing in p53 function, A549 (p53 wild type) and H1299 (p53 deficient type). The p53 status of A549 and H1299 cells were confirmed by examining p21^CIP1^ and hdm2 expressions, and CCK-8 assay revealed that there was no significant difference of DDP IC50 between DDP alone and DEX co-medication group in H1299 cells (Figure 4E). We also found in H1299 cells, DEX did not influence the SA-β-Gal activity (Figure 4F). A549 cells were transfected with p53 siRNA and control siRNA. Western blot for p53 confirmed suppression by p53 siRNA. As expected, p53 siRNA could further decrease SA-β-Gal activity (Figure 4G). Taken together, the results indicate that p53 can be activated by cytotoxic stress, and DEX co-treatment influence TIS is p53-dependent.

### NF-κB was an Important Mediator of DEX’s Role in Chemotherapy Insensitivity

**Figure 5:**
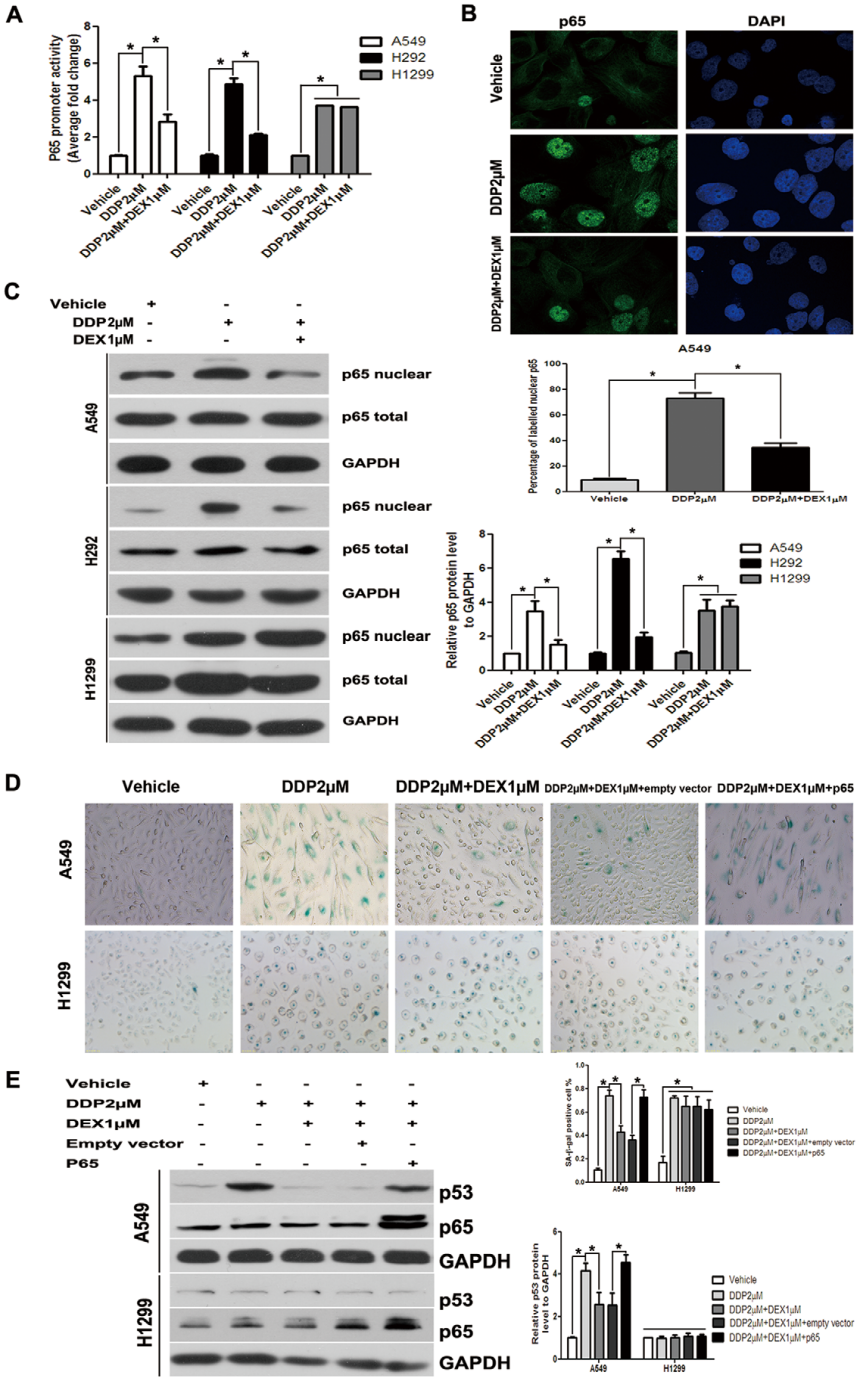
NF-κB is an important mediator of DEX’s role in chemotherapy insensitivity. (A) Cells were transiently transfected with NF-κB promoter-luciferase reporter plasmid. The luciferase activity in whole cell lysates was determined 24 hours post-transfection. Data shown are mean (± standard error of mean) of the ratio firefly luciferase activity over renilla luciferase activity and normalized to the ratio of vehicle group. (B) Cells were imaged 0.5 hours following DDP in the presence or absence of DEX. Nuclei of A549 were counterstained with DAPI. Vehicle group do have visible nuclei that illustrate the presence of NF-κB p65 in the nucleus. After DDP treatment, cells showed more translocation of NF-κB with visible nuclei demonstrating that NF-κB p65 is in the nucleus. (C) Translocation of p65 to the nucleus was measured by Western blot of nuclear exacts with an antibody against total p65 in A549, H292 and H1299 cells. (D) A549 and H1299 cells were transfected with pcDNA3.1-/p65 in combination with DDP in the presence of or absence of DEX. SA-β-gal activity was analyzed by microscopy at day 3 after drug washed. The percentage of SA-β-gal cells was presented in the histogram. (E) A549 and H1299 cells were transfected with pcDNA3.1-/p65 in combination with DDP in the presence of or absence of DEX, the expression of p53 was determined by Western-blot, equivalent protein loading between lanes was confirmed by Western analysis of GAPDH. Data represent means of six determinations ± standard error of mean (SEM). *p<0.05 and **p<0.01 analyzed by one-way analysis of variance (ANOVA).

To further characterize whether NF-κB signaling promotes or abrogates senescence, we determined NF-κB activity in NSCLC cells after DDP treatment with or without DEX. Treatment with DDP potently activated NF-κB promoter activity in A549, H292 and H1299 cells, and treatment with DEX decreased NF-κB promoter activity only in A549 and H292 cells (Figure 5A). Next, we detected released NF-κB molecules translocate to the nucleus following DDP treatment. DDP alone group showed nuclear translocation of NF-κB p65 and DEX co-treatment decreased the level of nuclear translocation (Figure 5B). The nuclear translocation of p65 was further confirmed by Western-blot. DDP treatment significantly promoted the nuclear localization of p65, whereas the total amount of p65 in cytoplasm was slightly changed based on the ratio of p65/GAPDH. And DEX co-medication decreased nuclear translocation in A549 and H292 cells but not in H1299 cells (Figure 5C).

To address the functional significance of NF-κB for TIS, we overexpressed p65 (pcDNA3.1/p65) in A549 and H1299 cells. In DEX co-treatment group, transfection of pcDNA3.1/p65, but not the empty control vector, resulted in a significant induction of p65 protein and p53 protein expressions in A549 cells rather than in H1299 cells (Figure 5E). DEX co-treatment group with transfection of pcDNA3.1/p65 also showed an increase of SA-β-Gal activity compared with that of DEX co-treatment with transfection of empty vector in A549 cells rather than in H1299 cells (Figure 5D). These results confirmed that NF-κB signaling pathways influenced DEX’s role through p53-dependent manner.

### DEX Co-treatment Reduced DDP Sensitivity *in vivo*

**Figure 6:**
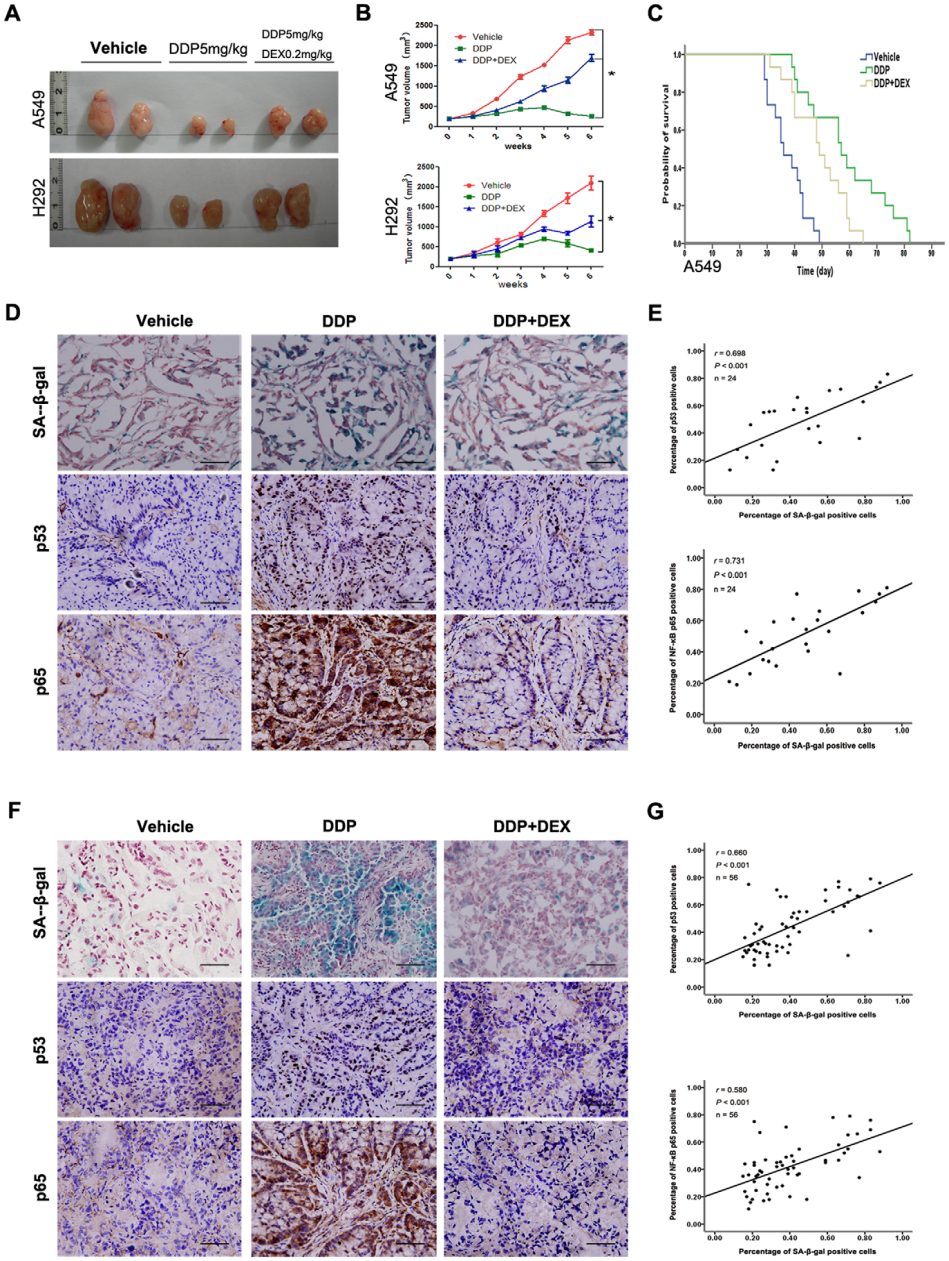
DEX co-treatment reduced DDP sensitivity *in vivo*. (A) The tumors in the nude mice (n = 15 in each group). A549 and H292 cells were subcutaneously injected into nude mice and tumors were harvested 6 weeks after treatment. (B) Tumor volume was measured weekly with a caliper and tumor volume (cm^3^) calculated. Tumor growth was significantly increased in DEX cotreatment group compared with DDP alone group. (C) For 42 days post-inoculation, mouse survival was monitored daily. Survival data were plotted on a Kaplan-Meier curve. Co-treatment with DEX significantly decreased survival when compared to DDP alone group. (D) Frozen sections were stained for SA-β-gal (blue) and paraffin sections were stained for p53 and NF-κB p65. (E) Correlation analysis between SA-β-gal and p53, NF-κB p65 expression in tumor of nude mice. (F) Representative SA-β-gal staining and immunohistochemistry p53 and NF-κB p65 staining (20×) in human lung tumor tissue. (G) Correlation analysis between SA-β-gal and p53, NF-κB p65 expression in NSCLC tissue. Data represent means of six determinations ± standard error of mean (SEM). *p<0.05 and **p<0.01 analyzed by one-way analysis of variance (ANOVA).

We next asked whether DEX co-treatment would also reduce chemotherapy sensitivity *in vivo*. A549 and H292 xenografts were grown in nude mice as models. At a tumor size of 200 mm^3^, DDP was injected i.p. weekly over a period of 6 weeks in the absence or presence of DEX, which was intragastric administration. Weekly measurement of the tumor volume revealed a profound reduction of tumor growth in DDP-treated mice at day 42, and DEX prevented the growth-inhibiting effect of DDP because the tumors grew faster in combination treatment (Figure 6A, 6B). Also, mice were treated with DDP with the presence or absence of DEX and their survival was monitored over a period of 6 weeks. DDP treated mice exhibited a significantly prolonged survival when compared to the control group mice. There was a 1.58 fold increase in the survival time (median survival time 57 days) of DDP treated mice, as compared to 36 days for the control group mice. Co-treatment with DEX condensed the prolonged median survival time to 49 days (Figure 6C). Furthermore, the number of SA-β-gal-positive cells in DDP-treated tumor dramatically increased, and DEX co-treatment group mice suppressed the increase caused by DDP (Figure 6D). Moreover, we found SA-β-gal activity was positively correlated with p53 and p65 expression (Figure 6D, 6E). These results demonstrated that DEX co-treatment reduced DDP sensitivity *in vivo*.

To further ascertain the influence of cellular senescence after DEX cotreatment in NSCLC *in vivo*, SA-β-gal staining and immunohistochemistry were carried out to analyze SA-β-gal activity and expressions of p53 and p65 in tumor tissues from 56 patients with NSCLC. Ethical approval was given by the medical ethics committee of Zhongshan Hospital, Fudan University. According to the different treatment, 56 patients were divided into 3 groups: patients without chemotherapy (n = 23), patients with primary DDP chemotherapy in the absence of DEX (n = 16) and patients with primary DDP chemotherapy in the presence of DEX (n = 17). SA-β-gal activity correlated positively with treatment but did not show association with any other clinicopathologic parameters (Table 1). Immunohistochemical analysis showed SA-β-gal activity was positively correlated with p53 and p65 expression in tumor tissues (Figure 6F, 6G). These results suggest that DEX cotreatment reduced DDP sensitivity positively correlated with cellular senescence.

**Table 1:** Correlation analysis between SA-β-gal activity and clinicopathological parameters in human NSCLC lung tissues.

Clinicopathological parameters	Patients (n = 56)	SA-β-gal expression		*P* value*
	Number	High (n = 19 )	Low (n = 37)	
***Age (years)***				0.645
≤51	23	7	16	
>51	33	12	21	
***Gender***				0.920
Male	30	10	20	
Female	26	9	17	
***ECOG Performance status***				0.492
0	33	10	23	
1	23	9	14	
***Histology***				0.757
Adenocarcinoma	34	11	23	
Squamous carcinoma	22	8	14	
***Histological grade***				0.784
Well and moderately differentiation	31	11	20	
Poorly defferentiation	25	8	17	
***TNM stage***				0.415
I,II	42	13	29	
III,IV	14	6	8	
***Chemotherapy***				0.001*
Without chemotherapy	23	2	21	
DDP without DEX	16	11	5	
DDP cotreatment with DEX	17	6	11	

Fifty-six patients with NSCLC were divided into SA-β-gal “high” group (whose final density was higher than the median) and “Low” group (whose final density was lower than the median.) The patient and disease profiles in each group were compared.

* *P*<0.05 is considered statistically significant (*t* test for continuous variables and *χ^2^* test for categorical variables).

## Discussion

In present study, we have investigated the influence of DEX co-treatment with chemotherapy on tumor growth, tumor cell viability, tumor migration and cellular senescence in NSCLC *in vivo* and *in vitro*. Although previous reports have demonstrated that DEX co-treatment could reduce several chemotherapy drugs sensitivity, its relationship with cellular senescence has not been previously demonstrated. We focused our tests of DEX co-treatment effects on the influence of therapy induced cellular senescence, which could provide equivalent or prolonged survival with fewer and less severe side effects related to cytotoxicity. We showed here that DEX co-treatment suppressed the effect of chemotherapy by blunting therapy-induced cellular senescence in NSCLC cell lines and tumor bearing mice. Furthermore, we also found DEX co-treatment influenced DNA damage signaling pathway, which played a core role in TIS. This course was in p53-dependent manner, and activation of NF-κB was associated with it. Thus, the above results indicate that GCs are highly suspicious to reduce sensitivity to cytotoxic therapy and need for carefully consideration on using it.

GCs have been demonstrated to protect normal tissue which may be good for patients, but the protection of cancer cells may impair the effect of chemotherapy [27]. Thus, it is understandable that the appeal for reevaluating the administration of GCs in the management of solid cancer patients has emerged recently. Recent studies suggested that DEX comedication in cancer therapy reduces chemotherapy sensitivity in the majority of cell lines derived from various malignancies including brain, breast, cervix, melanoma and neuroblastoma [3], [7]. Using cell counting, CCK-8 assay and Cell IQ detection, our results corresponded to these reports showing that DEX partially reverses chemotherapy effect not only in NSCLC cell lines but also in tumor xenografts. Also, there in contrast existed a study which focused on the head and neck cancer showing that DEX had no inhibition of the cytotoxic activity of cetuximab in cell lines [28]. The reasons for the findings may be related to intrinsic properties of specific tumor cells and suggested a more cell type-specific effect of glucocorticoids should be studied. Also, different response with DEX co-treatment according to different antineoplastic drugs may play a role. Moreover, wang [29] et al also demonstrated that pretreatment with DEX five days before antitumor therapy may increase activity of carboplatin and gemcitabine in mice bearing human cancer xenografts. This inconsistency of DEX effect may be related to the different delivery time of DEX, and also different methods of establishing human cancer xenograft models may play a role.

Cancer therapy has traditionally relied on cytotoxic treatment strategies on the assumption that complete cellular destructions of tumors. These approaches may produce complete cell death within a solid tumor and can cause severe side effects. An alternative strategy is the induction of TIS [30]. This approach to treatment could provide equivalent or prolonged survival with fewer and less severe side effects related to cytotoxicity and may provide a more realistic goal for the chronic management of some cancers [19], [31], [32]. Accumulating evidence now demonstrates that senescence has important part to play in the natural physiological response to tumor development [33], [34], [35]. Previous studies have shown that influence of drug effect by DEX co-medication was probably due to the influence of therapy induced apoptosis, but the association with TIS is largely unknown. For the first time, we here examined TIS in the presence or absence of DEX through characteristic morphological alterations of senescent cells, β-gal activity assay and SAHF formation, and we demonstrated that DEX protected tumors by the inhibition of TIS. This may be an important reason that can partially explain DEX co-treatment reduced chemotherapy sensitivity.

The question remains, how can we distinguish the cellular senescence from cellular apoptosis? A previous study has shown us the ability to distinguish between the stress levels required for senescence and apoptosis through MTT assay, flow cytometry analysis and DNA fragmentation [36]. Therefore, we used CCK-8 assay, an improved assay which is more effective than MTT, to detect cell viability with different concentration gradient of DDP. Thus we demonstrated the proper concentration of DDP (2×10^−6^ M) which can induce TIS rather than apoptosis. We also used TUNEL assay to confirm this effect. But seriously, we should know cellular senescence and cellular apoptosis are relative, and we couldn’t draw a clear line between them. In fact, effective dosing to achieve senescence will vary with the drug but may involve lower doses than those that generate apoptosis [37].

Following our appreciation of the fact that DEX co-treatment reduced chemotherapy sensitivity by inhibiting TIS, we next conducted additional experiments to understand its mechanisms. Chemotherapy drugs, such as DDP, can active DNA damage signaling pathway and disable normal cell-cycle checkpoint leading to growth arrest, senescence, and apoptosis [38], [39], [40]. In most cases, following DNA damage, activation of p53 led to induced expression of classical p53 targets including p21 and induced G1 arrest, thus the p53-dependent cellular senescence [41], [42]. Also, a recent study showed that down-regulation of a kind of acetyltransferase activity induced senescence by a mechanism that is independent of activation of p53 and p21^CIP1^ and resulted in a robust G2/M cell cycle arrest [43]. So, how does DEX influence the effect of chemotherapy, by p53-dependent manner or by p53-independent manner? Our studies showed that DDP can activate p53 signaling pathway and render cancer cells arrest in G1 phase, and as expected, DEX co-treatment attenuated the increase of p53 level. Therefore, it seems most likely that DEX modulated antineoplastic effect in a p53-dependent manner.

So far we have shown that DEX-mediated suppression of cellular senescence in NSCLC cells is attributable to the inhibition of p53 signaling pathway to some extent. Since the stress-activated signaling pathways, p53 and NF-κB, have a major role in the regulation of cellular senescence [44], [45], [46], and the p53 signaling pathway are inextricably linked to NF-κB signaling pathway [47], [48], [49], we next addressed whether DEX co-treatment influenced the activity of NF-κB. Our results showed that DEX co-treatment decreased NF-κB activity compared with DDP alone group. These were in accordance with previously published studies, which demonstrated that glucocorticoids (GCs) such as DEX often exert potent anti-inflammatory effects and could suppress NF-κB activity [50], [51]. Though some studies showed that in the NF-κB and p53 pathways where regulatory proteins act on both p53 and NF-κB with opposite functional consequences [52], p53 and NF-κB can not simply be divided into two solitary entities. In fact, crosstalk of p53 and NF-κB occurs at multiple levels and has to be considered as a highly context-specific event. Correspondingly, there is increasing evidence suggesting that NF-κB is necessary to induce accumulation of p53 and its signaling pathways [46], [53].

In conclusion, we show here that DEX co-treatment with chemotherapy can reduce drug sensitivity, which is due to the inhibition of cellular senescence to some extent. The mechanism to explain this insensitivity appears to involve in the decreased DNA damage and attenuation of G1 arrest level. Such changes are also in p53-dependent manner and NF-κB signaling pathway plays an important role in it. These findings are clinically relevant: low dose of DDP can induce cellular senescence rather than cellular apoptosis, which could provide equivalent survival with less severe side effects. DEX co-treatment with chemotherapy should be applied carefully in clinic.
